# Digital and Intelligent Rehabilitation Technologies in Stroke and Neurological Disorders: A Systematic Review of Artificial Intelligence, Virtual Reality, Gamification, and Emerging Therapeutic Platforms in Neurorehabilitation

**DOI:** 10.3390/bioengineering13020195

**Published:** 2026-02-09

**Authors:** Majeda M. El-Banna, Moattar Raza Rizvi, Waqas Sami, Ankita Sharma, Rushdy R. Atyeh

**Affiliations:** 1 College of Nursing, Health Sector, Qatar University, Doha P.O. Box 2713, Qatar; melbanna@qu.edu.qa; 2 Allied Health Science, Santosh Deemed to be University, Santosh Nagar, Ghaziabad 201009, Uttar Pradesh, India; dean.ahs@santosh.ac.in; 3 Department of Pre-Clinical Affairs, College of Nursing, QU-Health Sector, Qatar University, Doha P.O. Box 2713, Qatar; 4 Department of Physiotherapy, Amity Institute of Health Allied Sciences, Amity University, Noida 201313, Uttar Pradesh, India; asharma39@amity.edu; 5 School of Medicine, Jordan University of Science and Technology, Irbid 22110, Jordan; rushdyateyeh@yahoo.com

**Keywords:** artificial intelligence, neurorehabilitation, extended reality, wearable devices, systematic literature review

## Abstract

Artificial intelligence (AI), virtual reality (VR), gamification, and telerehabilitation are increasingly incorporated into neurorehabilitation to deliver adaptive, personalized, and remotely accessible interventions for individuals with stroke and other neurological disorders. These technologies aim to address key limitations in conventional rehabilitation by enhancing training intensity, patient engagement, accessibility, and real-time monitoring. This systematic review synthesizes evidence from clinical and simulation-based studies evaluating AI-assisted systems, non-AI gamified platforms, VR/exergames, telerehabilitation models, and simulation-driven architectures across neurological populations. A comprehensive search of PubMed, Scopus, Embase, CINAHL, and Web of Science (2010–2025) identified randomized controlled trials, pilot and quasi-experimental studies, telerehabilitation systems, VR/exergame interventions, AI-based adaptive tools, and computational or model-driven investigations, guided by a revised PICO framework. Data were extracted using a standardized template, with studies categorized by design, population, technological modality, and outcome domain. Risk of bias was assessed using validated tools, and GRADE was applied to stroke-specific clinical outcomes. Twenty-two studies met the inclusion criteria, encompassing both clinical trials and simulation/modeling research. Clinical studies reported improvements in motor function, balance, gait, swallowing, cognition, and psychosocial well-being, often accompanied by high usability and adherence. AI-enabled systems facilitated adaptive difficulty adjustment, automated feedback, and individualized progression, while non-AI platforms demonstrated strong engagement and meaningful functional gains. Simulation studies provided valuable insights into algorithm behavior, sensor-based modeling, and system optimization. Despite promising multi-domain benefits, methodological heterogeneity, limited long-term follow-up, and inconsistent AI transparency remain key challenges, underscoring the need for standardized outcomes, explainable AI, inclusive design, and robust multicenter trials.

## 1. Introduction

Stroke and other neurological conditions, such as traumatic brain injury, spinal cord injury, Parkinson’s disease, and multiple sclerosis, are still the major causes of long-term disability across the globe. Stroke in itself contributes to a huge share of global disability-adjusted life years [1,2]; however, analogous issues regarding access, severity, and persistence of rehabilitation are becoming increasingly reported in the context of the wider range of neurological conditions [3]. The challenges are further magnified in low- and middle-income countries (LMICs), with a lack of trained professionals, fragmentation of rehabilitation services, and a lack of socioeconomic equity being the underlying factors for delayed recovery and worse outcomes [4,5]. Increasing incidence rates and growing gaps in access to rehabilitation in India further highlight the necessity to develop scalable, technology-based strategies of neurorehabilitation that would be able to supplement conventional therapy [6].

Traditional neurorehabilitation is based on task-focused training accompanied by the involvement of a therapist aimed at achieving neuroplasticity and recovery of motor and cognitive functions [7]. Though efficient, such interventions are intensive in resources, geographically limited, and, in most cases, fail to provide the frequency and intensity necessary to achieve maximum recovery [8]. In addition, monotony, fatigue, and a lack of access to personal feedback might impede patient engagement, which does not, in turn, result in motivation impairment and inconsistent adherence to persistent challenges in neurological rehabilitation programs.

The digital rehabilitation technologies, such as telerehabilitation, virtual reality (VR), exergames, wearable sensors, and sensor-based feedback systems, have become scalable and flexible options that can be used to support therapy in both clinical and home settings [9]. These systems facilitate the immersive practice, real-time surveillance, and objective evaluation of performance, which reinforces continuity of care that is more than traditionally practiced in-person models. The recent literature shows that VR/game-based neurorehabilitation can be useful in enhancing motor, balance, and cognitive outcomes in neurological conditions [10,11]. In line with that, it has been reported that VR-based cognitive and physical training could improve neuroplasticity and functional outcomes of various neurological populations in a complementary manner [12,13].

Artificial intelligence (AI) adds another new dimension to flexibility because it allows movement analysis in real-time, predictive modeling, and custom therapy progression [14]. Machine learning, reinforcement learning (RL), and generative adversarial networks (GANs) assist in the continuous tracking of performance and dynamic adaptation of tasks to facilitate rehabilitation activities and to adapt automatically to the user’s ability and engagement level [15]. Combined with VR, augmented reality (AR), or serious games, AI-based personalization increases the feeling of immersion, motivation, and motor-learning effectiveness. The use of gamification (such as scoring systems, level progression, avatars, challenges, rewards, and instantaneous feedback) also enhances commitment and involvement in the treatment process [9,16]. Early implementations of adaptive, task-oriented digital environments, like the Rehabilitation Gaming System [17], and distributed systems, like RehabNet [18], have been shown to be viable in VR-based neurorehabilitation. More recent reviews are also optimistic about the potential of AI-driven rehabilitation systems to optimize neuroplasticity and motor recovery by adapting personalized, data-driven approaches to acquired brain injury populations [19], but recent feasibility studies also show that cognitive and motor rehabilitation tools supported by AI can be used in acquired brain injury populations [20].

Rehabilitation platforms based on AI are actively complemented with wearable sensors, motion-capture devices, and mobile apps that provide an uninterrupted stream of data that allows tracking the recovery patterns accurately and tailoring therapy according to individual needs [14]. These systems are able to vary the difficulty of tasks depending on patient-specific characteristics, such as impairment baseline, fatigue, and cognitive load, as well as engagement patterns. Even though robotic and AI-enhanced systems have proven beneficial [21,22], challenges associated with cost, complexity of the device, and scalability by clinicians remain. However, the literature on system-development indicates increasing viability of semi-autonomous and home-based AI-guided neurorehabilitation, and enhanced patient-friendly design and usability functionalities [20,23].

With the rapid growth of digital and smart neurorehabilitation technologies, an integrated synthesis is needed to examine how emerging tools are designed, categorized, and evaluated across neurological rehabilitation contexts. Existing reviews primarily focus on individual modalities, such as VR, telerehabilitation, or mobile applications, without jointly examining AI-driven systems, gamification strategies, and simulation-based platforms within a single analytical framework [24]. Accordingly, the purpose of this review is to provide a structured, system-level synthesis of neurorehabilitation technologies, encompassing AI-based and non-AI platforms, across stroke and other neurological conditions. This review adopts an ecosystem perspective to organize technologies by delivery setting, adaptive mechanisms, and outcome domains, thereby offering a conceptual framework to guide future research design, technology development, and evaluation. As a systematic review, this study does not generate primary clinical data; rather, its original contribution lies in the cross-domain evidence-mapping approach and the ecosystem-level conceptualization that integrates clinical outcomes, usability, and simulation/modeling evidence within one analytic structure. The primary outcomes of interest were motor function, balance and gait, swallowing, cognition, activities of daily living, usability and adherence, and psychosocial outcomes, and the certainty of evidence for stroke-specific clinical outcomes was assessed using GRADE.

## 2. Materials and Methods

### 2.1. Study Design and Protocol Registration

This systematic review followed the Preferred Reporting Items for Systematic Reviews and Meta-Analyses (PRISMA) 2020 guidelines to ensure methodological rigor, transparency, and reproducibility [25]. The protocol was prospectively registered in PROSPERO (Centre for Reviews and Dissemination, University of York, York, UK) under registration number CRD420251247889. It specified the review objectives, study design, eligibility criteria, data-extraction procedures, and planned synthesis methods, thereby reducing the potential for methodological bias.

### 2.2. Search Strategy

A comprehensive search of PubMed (U.S. National Library of Medicine, Bethesda, MD, USA), Scopus (Elsevier B.V., Amsterdam, The Netherlands), Embase (Elsevier B.V., Amsterdam, The Netherlands), CINAHL (EBSCO Information Services, Ipswich, MA, USA), and Web of Science (Clarivate, London, UK) was conducted for studies published between January 2010 and February 2025. Two reviewers (MEB and RRA) independently executed the search using structured combinations of MeSH and free-text terms related to stroke, cerebrovascular accident, artificial intelligence, machine learning, gamification, virtual reality, exergames, adaptive systems, simulation models, and telerehabilitation. Boolean operators were applied to refine the results, and the search was limited to studies published in English. Reference lists of included studies were screened to identify additional sources. Disagreements were resolved through discussion or adjudication by a third reviewer (MRR).

### 2.3. Eligibility Criteria

Studies were eligible if they evaluated digital, AI-driven, or technology-enhanced neurorehabilitation interventions involving adults (≥18 years). Eligible neurological populations included stroke, Parkinson’s disease, spinal cord injury, and multiple sclerosis. Simulation-based and model-driven studies related to neurorehabilitation system development were also included. Interventions were required to incorporate artificial intelligence, VR/AR, gamification, exergames, wearable sensors, mixed-reality platforms, or telerehabilitation, consistent with prior classifications of technology-assisted rehabilitation [3,26,27]. Eligible outcomes encompassed motor recovery, balance, gait, swallowing, cognition, activities of daily living, usability, adherence, psychological well-being, and engagement [9,28].

The PICO framework guided eligibility decisions. AI-based interventions were defined as those using machine learning, deep learning, neural networks, reinforcement learning, or predictive analytics capable of adaptive or autonomous therapeutic adjustment. Systems relying only on static rules or basic sensor-triggered feedback were categorized as digital but not AI-driven [3,27].

### 2.4. Screening and Study Selection

**Figure 1:**
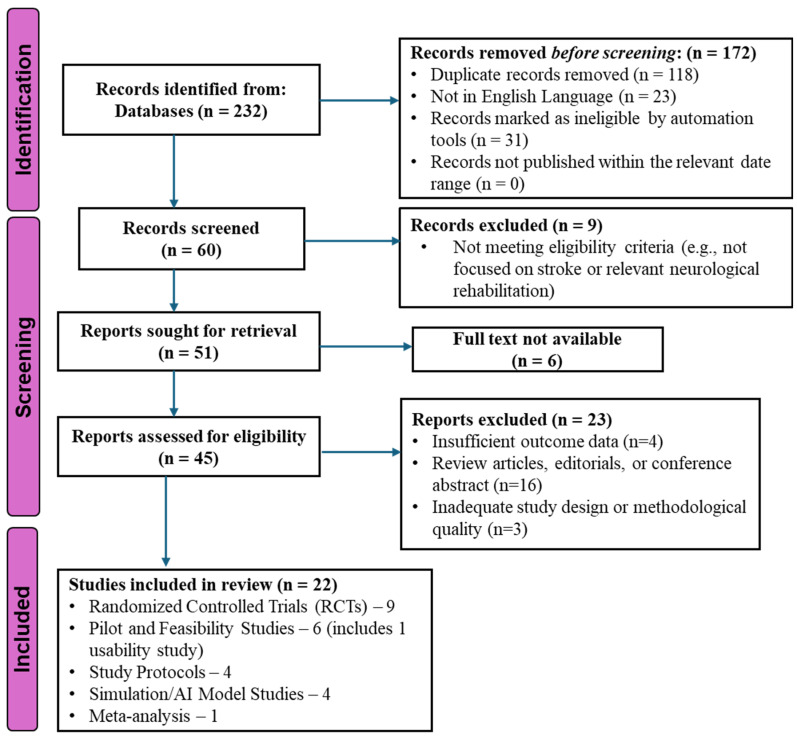
PRISMA 2020 Flow Diagram of Study Selection. This figure presents the PRISMA (Preferred Reporting Items for Systematic Reviews and Meta-Analyses) 2020 flow diagram depicting the study-selection process for this review. The search spanned January 2010 to March 2025 and included records identified through database searching and other sources. The diagram outlines the number of records identified, screened, excluded (with reasons), and ultimately included in the qualitative and/or quantitative synthesis. This structured flow ensures transparency in inclusion criteria and strengthens reproducibility.

All records were imported into EndNote 20 (Clarivate, London, UK) for deduplication and screening. Two reviewers independently screened all titles, abstracts, and full texts in duplicate, according to predefined eligibility criteria. Any discrepancies were resolved through discussion, with a third reviewer consulted when necessary. The screening process, including records identified, excluded, and retained, is presented in the PRISMA 2020 flow diagram (Figure 1) [25]. Inter-rater agreement during initial screening was 91%. Cohen’s κ was not interpreted because substantial class imbalance (few included vs. many excluded studies) can deflate κ despite high observed agreement.

### 2.5. Data Extraction and Classification

A standardized form was also used to extract data both independently and in duplicate. Inter-rater agreement between the reviewers of extracted variables was high to near perfect (κ = 0.80–0.94), and any discrepancies were sorted out through consensus or third-reviewer adjudication. A total of 22 studies were identified based on the existing frameworks that specify the relationship between technological modality and anticipated therapeutic and learning outcomes, such as home-based telerehabilitation systems, clinic-based systems, and simulation or AI-modeling studies [5,24,29].

### 2.6. Risk of Bias Assessment

Randomized controlled trials were evaluated using the Cochrane RoB 2 tool, assessing randomization, deviations from intended interventions, missing data, outcome measurement, and selective reporting [30]. Non-randomized studies were assessed using ROBINS-I. Certainty of evidence was evaluated with the GRADE approach. Simulation and protocol studies were described narratively and excluded from GRADE scoring [31].

### 2.7. Data Synthesis Strategy

There was great methodological and clinical heterogeneity, which excluded the presence of a meta-analysis; thus, an organized narrative synthesis was conducted in accordance with the existing guidelines [31]. The studies were classified and categorized as outcome domains, such as upper-limb functions, balance and gait, swallowing, cognition, usability, adherence, and AI-sponsored personalization. Differences in therapeutic effects were put in perspective by examining delivery models (home vs. clinic) and stroke chronicity (subacute vs. chronic).

## 3. Results

A total of 22 studies passed the eligibility criteria. High inter-rater agreement was observed during screening (91%). Table 1 presents a summary of study characteristics of nine randomized controlled trials (RCTs), six pilot or feasibility studies, four simulation or AI-modeling studies, and three intervention protocols. There were studies that were geographically varied, including Asia (n = 8), Europe (n = 7), North America (n = 4), and other regions (n = 3).

Considerable clinical and methodological heterogeneity was present across populations, intervention designs, and outcome measures. For clarity, studies were grouped into home-based telerehabilitation systems, clinic-based systems, and simulation-based or AI-driven models. The key digital platforms and rehabilitation systems evaluated across the included studies, together with their core applications and reported benefits, are summarized in Table 2. Across the included studies, follow-up durations ranged from immediately post-intervention to 3 months. Five studies reported post-intervention follow-up assessments, including 4-week follow-up after balance training [32], 1-month retention of motor and cognitive gains following VR-based rehabilitation [33], 3-month maintenance of upper-limb and ADLs improvements after combined VR and conventional therapy [34], follow-up functional assessments after multimodal robotic-VR rehabilitation [35], and 1-month follow-up demonstrating sustained swallowing and quality-of-life improvements after AI-based video-game therapy [36].

Among home-based telerehabilitation platforms, six studies incorporated gamification or AI-based adaptation. These systems generally demonstrated high usability and adherence while improving functional outcomes, such as upper-limb performance, swallowing function, and psychological well-being. One study reported a 71% completion rate and 95% usability score [37]. Another demonstrated strong usability and cost-effectiveness for a VR telerehabilitation program targeting balance [38]. A sensor-based home system produced motor gains comparable to clinic-based therapy [39], and an immersive VR platform resulted in clinically meaningful ARAT improvements in 84% of users [40]. Extended home deployment periods of up to 12 weeks were reported. Held (2018) implemented a 12-week unsupervised home-based VR intervention with a mean adherence rate of 71% and no serious adverse events [37]. Maggio (2023) delivered an 8-week fully remote cognitive telerehabilitation program with high session completion and no reported safety concerns [41]. Zhang (2025) evaluated a 4-week AI-based video-game intervention, reporting higher adherence in the intervention group (median 18 vs. 16 sessions) and no intervention-related adverse events [36]. Functional domains targeted by home-based systems are listed in Table 3.

Twelve clinic-based systems integrating VR, robotics, sensors, or AI demonstrated significant gains in upper-limb, lower-limb, and balance outcomes. A VR-assisted upper-limb program produced an 11-point FMA-UE improvement with a large effect size (η^2^ = 0.633, *p* < 0.001) [34]. An AI-enhanced multimodal system generated significant improvements in motor performance, Barthel Index, and Berg Balance Scale scores [42]. A gamified Wii Fit intervention produced larger Berg Balance Scale gains than conventional therapy (7.6 vs. 4.2 points; *p* = 0.004) [32]. Three clinic-based trials incorporated post-intervention follow-up assessments. Morone (2014) [32] evaluated balance outcomes at 4 weeks after completion of Wii-based training, Rodríguez-Hernández (2021) assessed upper-limb function and activities of daily living at 3 months following combined VR and conventional therapy [34], and Zhou (2022) conducted follow-up evaluations at 4, 8, and 20 weeks after robot-assisted VR rehabilitation [43], reporting maintenance of motor and balance gains. Additional clinic-based studies reported improvements in motor coordination, trunk stability, executive function, gait, and mood, demonstrating broad functional benefits across diverse technologies [33,35,44,45,46].

Several studies evaluated AI-driven or simulation-based rehabilitation frameworks. An AI dysphagia system improved GUSS, FOIS, and SWAL-QOL scores [36]. A motivational telerehabilitation system reduced mood disturbance and stress [47]. An adaptive home-based AI platform demonstrated feasible automated difficulty adjustment and high usability [48]. In clinical AI applications, Zhang (2002) reported higher adherence in the AI-based intervention group alongside statistically significant swallowing and quality-of-life improvements maintained at 1-month follow-up [49], while Lutokhin (2023) demonstrated high treatment adherence with sustained functional improvements following multimodal AI-assisted rehabilitation [35]. Four simulation-only studies showcased algorithmic innovations: a Bi-LSTM-Firefly model achieving >99% prediction accuracy [50], a GAN-based difficulty generator correlating strongly with real data (r = 0.74) [15], a reinforcement-learning model for adaptive reaching tasks [51], and a gamified BCI paradigm that increased engagement without altering classification accuracy [52].

**Figure 2:**
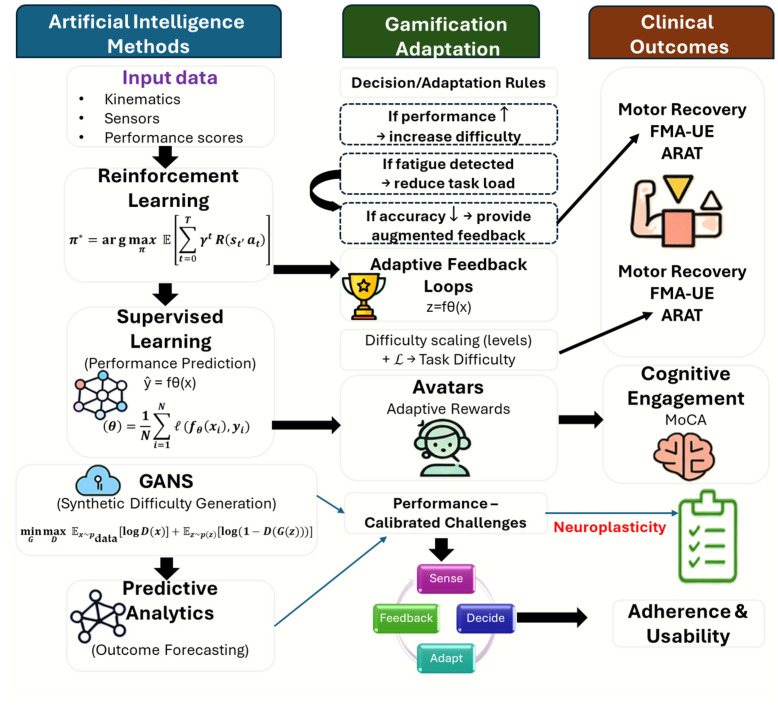
A conceptual illustration of adaptive decision-making, gamification adaptation, and associated clinical outcomes in digital neurorehabilitation systems. This figure illustrates how performance-related inputs (e.g., kinematics, sensor signals, and task scores) are processed through artificial intelligence-based learning paradigms to support adaptive personalization. Supervised learning mechanisms formalize performance prediction through loss minimization, reinforcement learning governs task progression via reward-based policy optimization, and generative adversarial models (primarily evaluated in simulation or in-silico environments) support automated generation and calibration of task difficulty. These algorithmic processes dynamically modulate gamification elements, such as feedback, difficulty scaling, rewards, avatars, forming closed feedback loops, that influence motivation engagement, therapy dose, and clinical outcomes, including motor recovery (e.g., FMA-UE, ARAT), cognitive engagement (e.g., MoCA), adherence, and usability. The mathematical expressions shown correspond to standard formulations described in the text and are presented as conceptual abstractions rather than implementation-specific or newly derived models. **↑** indicates improved performance leading to increased task difficulty, whereas **↓** indicates fatigue or reduced accuracy leading to task-load reduction.

AI and gamification features varied substantially. The AI-driven systems commonly used supervised learning, reinforcement learning, or predictive analytics to adjust difficulty and provide real-time feedback [39,42,50]. Gamification elements included challenge progression, scoring, rewards, avatars, and real-time feedback. Usability and engagement were consistently high across interventions, although measurement approaches differed. The conceptual relationships between artificial intelligence methodologies, gamification design features, and associated clinical outcome domains synthesized from the included studies are illustrated in Figure 2. A consolidated summary of study designs, sample sizes, and geographical distribution across the included literature is provided in Table 4.

Reporting of inclusivity-related considerations varied across the included studies. A subset of studies assessed cognition and visuospatial or perceptual status using global cognitive screening or attention- and neglect-oriented tasks [33,34], or targeted cognitive outcomes within digital rehabilitation programs [41]. However, explicit reporting of platform-level adaptations for users with cognitive, visual, or perceptual impairments was uncommon. Usability and user-experience reporting most frequently emphasized general satisfaction, clarity of rules, and visual attractiveness [52], while accessibility-oriented design features such as adaptable visual feedback, reduced cognitive load, or impairment-specific interaction modes were infrequently described.

An overview of the certainty of evidence across outcome domains, assessed using the GRADE framework, is presented in Table 5. Certainty of evidence (GRADE) was moderate for upper-limb motor outcomes and for balance and gait, low for ADLs, cognition, engagement, and quality of life, and very low for usability and adherence. Downgrading was primarily driven by short follow-up durations (≤3 months in most trials) and incomplete reporting of long-term outcomes in 17 of 22 studies [32,53]. Only stroke-specific clinical studies informed GRADE scoring; VR-only studies, protocols, simulation models, and non-stroke neurological samples were synthesized narratively.

**Table 1:** Summary of included studies on AI-based, VR-based, gamified, and simulation-supported rehabilitation systems.

Author, Year	Study Design	Objective and Population	Intervention	Outcome Measures	Results	Conclusion	AI Method/ Gamification Feature
Rodríguez-Hernández et al., 2021 [34]	RCT (Clinical)	To assess the effect of VR-based therapy on upper-limb motor function in patients with strokes (N = 43)	Conventional therapy + VR exposure therapy vs. conventional therapy alone for 4 weeks	Fugl-Meyer Assessment-Upper Extremity (FMA-UE), Modified Ashworth Scale, Stroke Impact Scale 3.0	Significant improvement in FMA-UE (~11-point increase), η^2^ = 0.633, *p* < 0.001	VR enhances traditional therapy and demonstrates a large effect size	Immersive VR environment with real-time performance feedback and adaptation
Kang et al., 2023 [54]	Protocol (Planned RCT–Clinical)	To evaluate home-based VR exergame training for post-patients with strokes (N = 120 planned)	8 weeks of home-based VR exergame vs. daily life activity (control)	Endurance, strength, ADLs, gait, QoL	Pending-protocol stage	Home-based VR is expected to improve community-level rehabilitation outcomes	Home VR exergaming with scoring and progressive difficulty (telerehabilitation)
House et al., 2016 [44]	Pilot Study (Clinical Feasibility)	To evaluate team-based gamified VR rehabilitation for chronic stroke in nursing homes (N = 23)	BrightArm Duo VR using robotic-enabled workstation; collaborative competition with remote participants	ROM (18/23 variables), task completion time, engagement, depressive symptoms	18/23 ROM variables improved significantly (*p* = 0.01)	Team-based gamified VR is feasible and beneficial in nursing home settings	Robot-assisted VR; multiplayer collaboration/competition; score feedback
Zhou et al., 2022 [43]	Protocol (Robot-Assisted Clinical Trial-In Progress)	To assess NeuCir-VR combined with robotic lower-limb rehabilitation (N = 40 planned)	Robot assistance + NeuCir-VR vs. robot + standard VR, 5 sessions/week for 4 weeks	FMA-LE, Berg Balance Scale, fMRI, Modified Ashworth Scale	Pending	NeuCir-VR expected to promote neuroplasticity and balance recovery	Robotic assistance + neural-circuit VR training framework
Bai et al., 2022 [42]	RCT (AI-Integrated Clinical Intervention)	To compare an AI-enhanced VR rehabilitation system with medication-only controls in patients with strokes (N = 50)	AI-VR personalized adaptive therapy for 10 weeks	FMA-UE, FMA-LE, FTHUE-HK, Barthel Index, Berg Balance Scale	Significant improvements in all motor outcomes (*p* < 0.05); Barthel Index ↑ ~25 points	AI-driven personalization improves functional recovery across domains	AI-adaptive VR tasks with continuous monitoring and feedback
Morone et al., 2014 [32]	RCT (Gamified Clinical Intervention)	To examine Wii Fit gamified balance therapy in subacute stroke (N = 50)	Wii Fit + PT vs. balance therapy + PT	BBS, Barthel Index, 10 MWT, FAC	BBS ↑ 7.6 vs. 4.2 (*p* = 0.004); BI ↑ ~22.8 points	Low-cost gamified balance therapy can enhance post-stroke recovery	Commercial games (Wii Fit) with goal-oriented balance tasks
Chen et al., 2022 [55]	Meta-analysis (43 RCTs)	To evaluate overall effects of VR-supported UL rehabilitation	VR-based therapy vs. conventional therapy	FMA-UE, ROM, strength, FIM, QoL	SMD values: UE = 0.45; ROM = 1.01; strength = 0.79 (all *p* < 0.001)	VR-supported therapy is effective for upper-limb recovery across trials	Adaptive VR motor-learning environments
Ahmed et al., 2020 [56]	Protocol (Immersive VR RCT-Planned)	To test immersive VR for upper-limb rehabilitation in ischemic stroke (N = 262)	Task-oriented multisensory rehabilitation (TMSR) + immersive VR vs. TMSR	FMA-UE, UK FIM-FAM	Pending	Immersive VR expected to enhance early subacute motor rehabilitation	Fully immersive 3-D VR environment with structured progression
Faria et al., 2018 [33]	RCT (Clinical Cognitive–Motor)	To test Reh@Task VR platform for cognitive-motor rehabilitation in chronic stroke (N = 24)	Reh@Task + OT vs. OT alone	MoCA, Bell’s test, Digit Cancellation, FMA-UE, Barthel Index	Greater improvements in FMA-UE and cognitive measures	Combined cognitive-motor VR provides additional benefit beyond OT	Gamified VR dual motor-cognitive tasks with adaptive performance tracking
Myung-Mo Lee et al., 2016 [45]	Pilot Study (Clinical Usability)	To evaluate VR canoe game for trunk stability and upper-limb function (N = 10)	VR canoe game 30 min, 3×/week for 4 weeks + PT	Trunk stability, balance, UL coordination, SUS	All outcomes improved; high usability scores	VR canoe-based therapy is feasible and supports motor/postural improvement	Game-based dynamic trunk control; high usability ratings
Maggio et al., 2023 [41]	RCT (Cognitive Telerehabilitation–MS)	Evaluate VR cognitive telerehabilitation in multiple sclerosis (N = 36)	VRRS-based cognitive TR (Khymeia Group, Padova, Italy)	MSQoL-54	Mental QoL ↑ 20.5 points (*p* < 0.001)	VR cognitive TR improves mental QoL	VRRS cognitive platform with interactive session tasks
Lutokhin et al., 2023 [35]	RCT (Exoskeleton + FES + VR)	Evaluate combined robotic, FES, and VR rehabilitation for early ischemic stroke recovery (N = 130)	Exoskeleton + FES + VR vs. comparators	Tinetti scale, muscle strength, stabilometry	Balance ↑ 7.1; gait ↑ 6.4; strength ↑ 13.6%	Multimodal VR-robotic systems yield strong early recovery benefits	Robotic gait + FES with VR-enhanced feedback
Ali et al., 2023 [46]	RCT (Parkinson’s-Gamified VR)	Compare VR vs. conventional PT for balance and QoL (N = 46)	VR balance/motor rehabilitation	SF-36, Barthel Index, BBS	BI ↑ 11 points; BBS ↑ 5.8 (*p* < 0.05)	VR improves balance and QoL in Parkinson’s	Gamified VR tasks with rewards and feedback
Paul et al., 2024 [57]	Protocol (VR-cRGS RCT)	To test VR-cRGS for stroke upper-limb recovery (N = 162 planned)	VR-cRGS vs. PT	FMA-UE, WMFT, Barthel Index, SF-36	Pending	VR-cRGS may improve upper-limb outcomes	Mirror-based VR gaming; feedback-based movement control
Lülsdorff et al., 2023 [40]	RCT (Immersive VR)	Compare immersive VR vs. robotic electromechanical training (N = 52)	CUREO (iVR) (CUREosity GmbH, Düsseldorf, Germany) vs. ARMEOSpring + therapy	ARAT, UEQ	ARAT ↑ 9.8 vs. 5.1; 84% vs. 50% achieved MCID	iVR may be equal or superior to robotic training	Immersive VR with real-time arm tracking
Held et al., 2017 [37]	Pilot (Home-Based VR TR)	Evaluate home-based VR telerehabilitation (N = 15)	REWIRE VR platform, 12-week balance exergames	Compliance, usability, adverse events	71% completion; 95% TAM usability; no adverse events	Safe, feasible, effective home VR system	Gamified VR balance tasks; telerehab monitoring
de Castro-Cros et al., 2020 [52]	Pilot (BCI-Partially Simulated)	Evaluate gamified vs. non-gamified BCI rehabilitation (N = 16)	BCI + avatar/FES vs. BCI without gamification	Classification accuracy, user satisfaction	Accuracy similar, engagement higher with gamification	Gamification boosts engagement in BCI rehab	Avatar-based BCI with reward structure
Alsheikhy et al., 2025 [50]	Simulation (In silico AI Model)	Develop personalized VR stroke therapy using Bi-LSTM + Firefly	AI-driven adaptive VR system	Prediction accuracy, task success	99.06% accuracy; 98% task success; task duration ~50 s	Strong AI potential for personalized VR	Bi-LSTM + Firefly optimization
Pelosi et al., 2024 [51]	Simulation (reinforcement learning)	RL-driven VR reaching-movement adaptation	Q-learning-based bubble-reaching VR system	Spatial adaptation performance	Effective adaptation across sessions; works for 2 participants	RL may support autonomous difficulty progression	Reinforcement learning for spatial cue modification
Zhang et al., 2025 [36]	RCT (AI–Gamified Dysphagia Rehab)	Evaluate AI-video game swallowing therapy post-stroke (N = 84)	AI-VG with lip, tongue, CTAR exercises	GUSS, SSA, FOIS, MNA-SF, SWAL-QoL, adherence	GUSS ↑ 4.02; FOIS ↑ 1.07; adherence ↑ (18 vs. 16 days)	AI-based gamified dysphagia rehab is effective	AI adaptive difficulty + gamified swallowing tasks
Burdea et al., 2021 [48]	Usability Study	Evaluate AI-adaptive BBG controller + BrightBrainer VR	VR games + AI controller	Error rate, completion, USE scale	Usability 6–7/7; difficulty scaling worked as intended	AI-adaptive controller is feasible and usable	Automatic difficulty adaptation
Chen et al., 2024 [15]	Simulation (GAN-Based)	Develop GAN-based difficulty-modulation engine for rehab games	GAN model (“Egg Catcher”)	Pearson r, training loss, variation, convergence	Pearson r = 0.74; 4.5× less variation; faster convergence	GANs promising for auto-tuning difficulty	GAN-based difficulty engine

Note: RCT: randomized controlled trial, VR: virtual reality, UE: upper extremity, LE: lower extremity, FMA: Fugl–Meyer Assessment, ADLs: activities of daily living, QoL: quality of life, ROM: Range of Motion, CTAR: Chin Tuck Against Resistance, GUSS: Gugging Swallowing Screen, SSA: Standardized Swallowing Assessment, FOIS: Functional Oral Intake Scale, MNA-SF: Mini Nutritional Assessment-Short Form, SWAL-QoL: Swallowing Quality of Life, SUS: System Usability Scale, MSQoL-54: Multiple Sclerosis Quality of Life-54, 10 MWT: 10-Meter Walk Test, ARAT: Action Research Arm Test, TAM: Technology Acceptance Model, BCI: brain–computer interface, FES: functional electrical stimulation, GAN: generative adversarial network, LSTM: Long Short-Term Memory, Bi-LSTM: Bidirectional Long Short-Term Memory, USE: Usefulness, Satisfaction, and Ease of Use Questionnaire, TR: telerehabilitation, VRRS: Virtual Reality Rehabilitation System, iVR: immersive virtual reality, RL: reinforcement learning, UL: upper limb, PT: physical therapy, TMSR: task-oriented multisensory stimulation rehabilitation, FIM: functional independence measure, and UK FIM-FAM: United Kingdom Functional Independence Measure and Functional Assessment Measure.

**Table 2:** Key platforms used across AI-Based, gamified, and VR-supported stroke rehabilitation.

Author, Year	Platform	Description	Examples of Application	Benefits	Key Insights
Rodríguez-Hernández et al., 2021 [34]	VR exposure therapy	Interactive VR therapy environment combined with conventional rehab	Upper limb function, tone, stroke recovery	Enhanced motor function and recovery	VR augments traditional therapy; high effect size (η^2^ = 0.633)
House et al., 2016 [44]	BrightArm Duo system (Bright Cloud International Corp., North Brunswick, NJ, USA)	Robotic table + VR team-based gaming	Upper-limb ROM, motivation, depression	Improved ROM, enjoyment, and compliance	Gamified teamwork model feasible in nursing homes
Bai et al., 2022 [42]	AI-VR rehab system	Game-based rehab guided by AI system for stroke	Motor scores, ADLs, balance	Significant gains in FMA and Barthel Index	AI-driven personalization improves recovery
Morone et al., 2014 [32]	Nintendo Wii Fit (Nintendo Co., Ltd., Kyoto, Japan)	Commercial gaming system adapted for stroke rehab	Balance training in subacute stroke	Superior gains in BBS and ADL vs. standard therapy	Low-cost, accessible game-based therapy works
Faria et al., 2024 [58]	Reh@Task	VR cognitive–motor dual-task training platform	Cognition, motor, ADLs	Better arm recovery and cognitive gains	Dual-targeted VR intervention is effective
Myung-Mo Lee et al., 2016 [45]	Canoe Game-based VR	Trunk postural training using a canoe-themed VR interface	Trunk stability and upper-limb motor control	Usability confirmed; improved stability and function	Novel VR settings like canoe are engaging and effective
Lülsdorff et al., 2023 [40]	CUREO (immersive VR)	Immersive virtual reality system for upper-limb rehab	Motor recovery, user experience	Comparable or superior to robotic therapy	iVR is clinically effective and better accepted
de Castro-Cros et al., 2020 [52]	Gamified BCI + FES	Brain–computer interface linked to functional electrical stimulation and gamified avatar	User satisfaction, stroke recovery	High engagement, preserved accuracy	Gamification enhances BCI-based rehab usability
Burdea et al., 2021 [48]	BrightBrainer BBG system	AI-adaptive game controller for home-based VR rehab	Task adaptation, usability testing	Highly usable, customizable rehab tool	AI improves user-level personalization in telerehab
Chen et al., 2024 [15]	GAN-based Adaptive Difficulty Planner	AI model to generate personalized rehab task difficulty levels for stroke therapy games	Adaptive game difficulty in upper-limb rehab simulations	Reduces training loss and difficulty variance; generalizes well across demographics	Automates personalization of task difficulty, enabling scalable game design
Zhang et al., 2025 [36]	AI-based Gamified Swallowing System	Tablet-based gamified rehab with AI-driven feedback for lips, tongue, and CTAR training	Post-stroke dysphagia therapy	Improves swallowing function, oral intake, QoL, and adherence	First AI-gamified platform targeting dysphagia with high satisfaction and effectiveness

Note: VR—virtual reality, ROM—Range of Motion, ADLs—activities of daily living, FMA—Fugl–Meyer Assessment, BBS—Berg Balance Scale, AI—artificial intelligence, BCI—brain–computer interface, FES—functional electrical stimulation, BBG—BrightBrainer Game controller, GAN—generative adversarial network, CTAR—Chin Tuck Against Resistance, QoL—quality of life.

**Table 3:** Reported outcomes across AI-based, gamified, and VR-supported rehabilitation.

Outcome	Description	Examples of Application	Representative Quantitative Outcomes	Benefits	Key Insights
Upper-Limb Motor Recovery [34,58,59]	Improvement in arm and hand function using gamified systems	VR therapy, BrightArm Duo, NeuroAlreh@b	FMA-UE + 9–11 pts (*p* < 0.05); η^2^ = 0.63; ARAT + 9.8 pts; Adherence ≥ 85%	Enhanced FMA scores, ROM, coordination, and functional independence	Gamification appears to support motor learning and adherence
Balance and Gait Improvement [32,35,37]	Recovery of postural control and walking through VR or robotic games	Wii Fit, Exoskeleton+ FES + VR), REWIRE	BBS + 5–8 pts; Tinetti + 6–7 pts (*p* < 0.05); Adherence > 80%	Improved BBS, Tinetti scores, reduced fall risk	Interactive balance games were generally well tolerated and may support balance improvement at home
Cognitive Engagement and Compliance [40,48,52]	Patient motivation and sustained use of VR or AI platforms	BBG System, BCI + FES, iVR	SUS > 85%; TAM 95%; USE 6–7/7 scale	High usability ratings, engagement scores, sustained task repetition	Gamified telerehab is well accepted and may reduce dropout rates
Swallowing Function [36]	Gamified AI-based therapy for post-stroke dysphagia rehabilitation	AI-VG exercises for lips, tongue, CTAR	GUSS + 4.0; FOIS + 1.1 (*p* < 0.001); Adherence ≈ 90%	Improved GUSS, FOIS, and SWAL-QOL scores; higher adherence and satisfaction	Gamified telerehab shows encouraging results for specialized domains such as dysphagia
Personalization and Adaptive Training [15,50]	AI-driven systems that adjust rehab tasks in real-time	GAN difficulty design, Bi-LSTM Firefly system	Accuracy 98–99%; r = 0.74 vs. real data	Better matching of task to user ability, faster progress	Generative and predictive AI tools show potential to enhance self-guided telerehab precision

Note: FMA—Fugl–Meyer Assessment, ROM—Range of Motion, BBS—Berg Balance Scale, FES—functional electrical stimulation, BBG—BrightBrainer Game controller, BCI—brain–computer interface, iVR—immersive virtual reality, AI—artificial intelligence, CTAR—Chin Tuck Against Resistance, GUSS—Gugging Swallowing Screen, FOIS—Functional Oral Intake Scale, SWAL-QOL—Swallowing Quality of Life, GAN—generative adversarial network, Bi-LSTM—Bidirectional Long Short-Term Memory.

**Table 4:** Summary of included studies by design type, sample size, and studies included.

Study Design	No. of Studies	Sample Size (Mean ± Range)	Regions Represented (with No. of Studies)
Randomized Controlled Trials (RCTs)	9	≈515 participants (68 ± 35; 24–130)	Europe (4): [34,40,41,58]
			Asia (3): [32,36,42]
			Middle East (1): [46]
			North America (1): [35]
Pilot Studies	4	≈64 participants (16 ± 6; 10–23)	Europe (2): [45,52]
			Asia (1): [37]
			North America (1): [44]
Protocols (Registered/Ongoing)	4	≈584 planned (181 ± 73; 120–262)	Asia (2): [43,54]
			Europe (1): [57]
			Multinational (1): [56]
Meta-Analysis/Systematic Review	1	43 RCTs pooled (N = 1893)	Global/Multinational: [55]
Simulation/AI Model Studies	3	Not applicable (Simulated datasets)	Asia (2): [15,50]
			Europe (1): [51]
Usability Studies	1	N = 2	North America (1): [48]
Total	22 studies	≈3129 participants (clinical + simulated)	Europe (9), Asia (7), North America (3), Middle East (1), Multinational (2)

**Table 5:** Summary of certainty of evidence (GRADE) across outcome domains.

Outcome/Domain	No. of Studies (Designs)	Risk of Bias (1–4)	Inconsistency (1–4)	Indirectness (1–4)	Imprecision (1–4)	Publication Bias (1–4)	Mean Score	Overall Certainty (GRADE)
Upper-limb Motor Function	11 (8 RCTs, 3 pilots)	3	3	4	3	4	3.4	Moderate
Balance and Gait	4 RCTs	3	3	4	2	4	3.2	Moderate
Swallowing Function	1 RCT	4	2	4	2	4	3.2	Low
Activities of Daily Living (ADLs)	2 RCTs	3	2	4	2	4	3.0	Low
Cognition/Engagement	3 (2 RCTs, 1 pilot)	2	2	4	2	4	2.8	Low
Usability/Adherence	5 pilot or feasibility studies	2	2	2	1	4	2.2	Very Low
Quality of Life/Psychosocial Well-being	3 RCTs	3	2	4	2	4	3.0	Low
Overall Summary of Evidence	22 studies (9 RCTs, 6 pilots, 4 simulations, 3 protocols)	-	-	-	-	-	≈3.0	Moderate → Low overall certainty

Note: Each GRADE domain is rated from 1 (very serious limitation) to 4 (no limitation). Only the studies involving stroke participants were graded for certainty; simulation models, protocols, and non-stroke trials were summarized narratively but excluded from domain scoring. The risk of bias was primarily rated using the Cochrane RoB 2.0 tool; pilot and feasibility designs were automatically downgraded one level for design limitations. Inconsistency was downgraded when substantial variation existed in intervention type, delivery setting, or outcome measurement. Imprecision reflects small sample sizes and wide variability across study designs and outcome definitions. Publication bias could not be reliably assessed due to the limited number of eligible RCTs per outcome.

**Figure 3:**
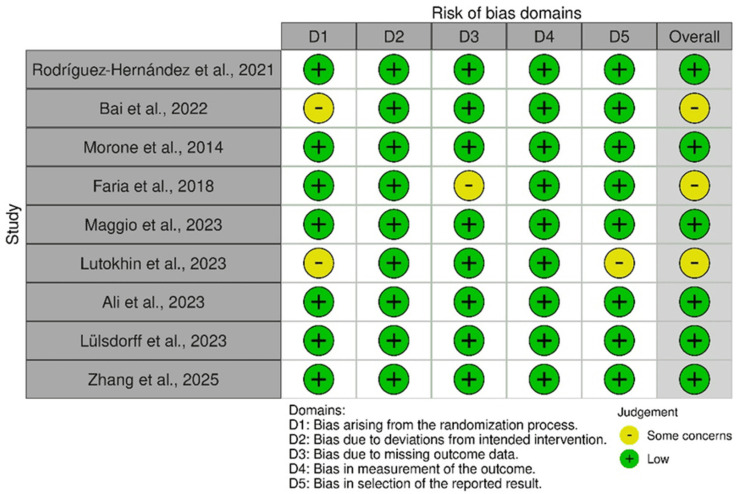
Cochrane Risk of Bias (RoB 2.0) Traffic-Light Plot for Included Randomized Controlled Trials [32,33,34,35,36,40,41,42,46]. This traffic-light plot displays the domain-level risk-of-bias assessments for each randomized controlled trial (RCT) included in this review, evaluated using the Cochrane Risk of Bias 2.0 (RoB 2.0) tool. Each column represents a study, and each row corresponds to one of five domains: (1) bias arising from the randomization process, (2) deviations from intended interventions, (3) missing outcome data, (4) measurement of the outcome, and (5) selection of the reported result. The color coding summarizes the individual judgments for each domain.

**Figure 4:**
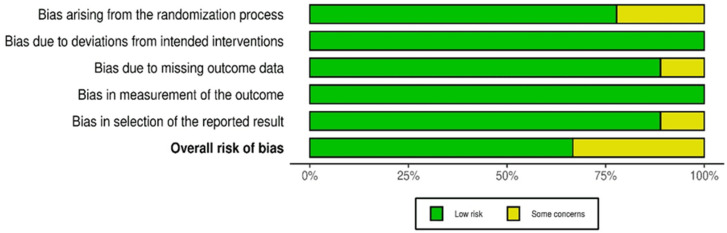
Domain-Level Summary Plot of Risk of Bias Across Randomized Controlled Trials (RoB 2.0). This summary bar plot illustrates the distribution of risk-of-bias assessments across the five RoB 2.0 domains for all the included RCTs. For each domain, the bars indicate the proportion of studies classified as at a low risk, some concerns, or high risk of bias. This visual summary provides a comprehensive overview of the methodological quality and identifies areas that may affect the strength and interpretation of the findings.

Risk of bias assessments are shown in Figure 2, Figure 3 and Figure 4. Seven RCTs were rated low risk overall, while two showed some concerns related to randomization or protocol deviations [35,42]. Most trials had minimal missing data and used validated outcome measures. Non-randomized studies exhibited moderate to serious risk of bias due to design limitations, small samples, and incomplete follow-up, contributing to GRADE downgrading.

**Figure 5:**
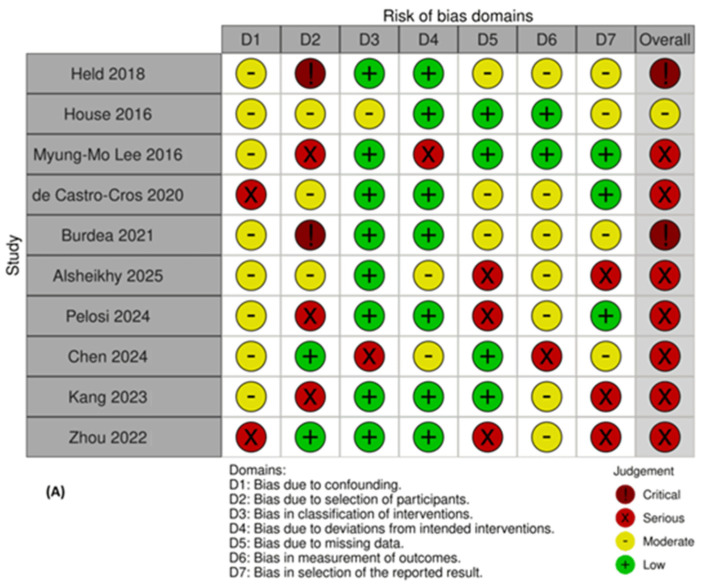

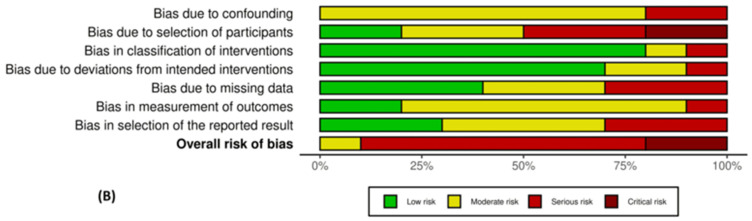
The risk of bias assessment for non-randomized studies using the ROBINS-I tool. (**A**) A traffic-light plot showing domain-specific judgements for each non-randomized study assessed using the ROBINS-I (Risk of Bias in Non-randomized Studies of Interventions) framework. Each column represents a study, and each row corresponds to a ROBINS-I bias domain: confounding, participant selection, classification of interventions, deviations from intended interventions, missing data, outcome measurement, and selection of reported results. (**B**) A summary plot displaying the proportion of studies rated at each risk level across the ROBINS-I domains. Together, the plots demonstrate that most non-randomized studies exhibited moderate-to-serious risks of bias, mainly due to the absence of randomization, small sample sizes, and incomplete follow-up reporting [22,29,31,37,38,39,40,41,54,55].

Across the included studies, artificial intelligence-driven systems commonly operated through adaptive decision-making pipelines that integrated performance data such as kinematics, sensor signals, and task scores to inform real-time task adaptation and feedback [36,42,48]. Supervised learning, reinforcement learning, predictive analytics, and generative models were used to support performance prediction, difficulty adjustment, and outcome forecasting, either within clinical interventions or simulation-based frameworks [15,35,50]. These algorithmic processes were frequently coupled with gamification features, including adaptive feedback loops, scoring or levels, rewards, avatars, and performance-calibrated challenges, which were associated with clinical outcome domains, such as motor recovery, cognitive engagement, adherence, and usability [33,37,52]. These algorithmic components, data inputs, and decision outputs are summarized in Table 6. Figure 5 synthesizes the reported AI methodologies, gamification adaptation logic, and associated clinical outcome domains across the included literature.

The adaptive behavior observed across the digital neurorehabilitation systems is grounded in a shared set of mathematical principles that govern learning, decision-making, and task adaptation. Articulating these principles provides a unifying lens through which otherwise heterogeneous AI-driven and non-AI digital interventions can be interpreted within a common analytical framework. Standard mathematical formulations from supervised learning, reinforcement learning, and generative modeling formalize how performance data are mapped to predictions, how task parameters are optimized over time, and how individualized training conditions are generated. By making these foundations explicit, the review strengthens conceptual coherence across technological modalities and clarifies the mechanistic pathways through which adaptive digital systems influence engagement, training dose, and functional outcomes in neurorehabilitation.

Formally, many of the adaptive mechanisms reported across the included studies can be described using well-established mathematical formulations that originate from machine learning theory. In supervised learning–based systems, adaptive feedback or difficulty adjustment is commonly derived from minimizing a loss function that maps observed performance features to predicted outcomes [60]:

$$L(\theta )=\frac{1}{N}\sum_{i=1}^{N}\ell (f_{\theta }(x_{i}),y_{i})$$

where $x_{i}$ represents input features, such as kinematic or sensor-derived performance metrics, $y_{i}$ denotes observed or target performance outcomes, $f_{\theta }\left(\cdot \right)$ is a parameterized model, and $\ell \left(\cdot \right)$ is a task-specific loss function. Variants of this formulation underpin AI-assisted rehabilitation systems that adapt task parameters based on movement accuracy, error rates, or task completion metrics, as reported in several included clinical studies [42,48].

Adaptive decision-making in reinforcement learning–driven rehabilitation environments can be expressed through optimization of an expected cumulative reward function [61]:

$$\pi^{*}=arg\;\underset{\pi }{max}\;E\left[\sum_{t=0}^{T}\gamma^{t}R(s_{t},a_{t})\right]$$

where π denotes a policy governing task selection or difficulty progression, $R(s_{t},a_{t})$ represents a reward function reflecting performance quality or engagement, and γ is a discount factor. This formulation provides the theoretical basis for systems that dynamically adjust task difficulty or feedback in response to real-time performance trajectories, as observed in adaptive VR-based and home-based rehabilitation platforms included in this review [36,51].

Generative modeling approaches employed in simulation-based rehabilitation studies are commonly formalized using a minimax optimization objective, as in generative adversarial networks (GANs) [62]:

$$\underset{G}{min}\;\underset{D}{max}\;E_{x∼p_{\mathrm{data}}}[logD(x)]+E_{z∼p(z)}[log(1-D(G(z)))]$$

where the generator G produces individualized task parameters or difficulty levels and the discriminator D evaluates their consistency with observed performance distributions. Such formulations support automated personalization of rehabilitation tasks and were primarily reported within simulation and proof-of-concept studies included in this synthesis [15,50].

Together, these mathematical abstractions provide a formal lens for understanding how adaptive digital rehabilitation systems translate performance data into personalized training trajectories, thereby linking algorithmic design choices to engagement, therapy dose, and functional recovery outcomes across heterogeneous intervention platforms [63].

## 4. Discussion

This systematic review offers an ecosystem-level, system-based synthesis of digital neurorehabilitation technologies by integrating clinical outcomes, usability evidence, and simulation/modeling findings across AI-driven and non-AI gamified platforms. By jointly considering technological design features, delivery setting, and adaptive mechanisms alongside outcome domains, this review advances an integrating framework that extends prior modality-focused syntheses. The included interventions span AI-based systems, non-AI gamified platforms, VR/AR applications, exergaming tools, and sensor-supported telerehabilitation. These findings are interpreted in the context of established stroke rehabilitation principles, emphasizing task-specific training, adequate intensity, repetition, and neuroplasticity, as articulated in contemporary clinical guidelines [64]. Taken together, this ecosystem-oriented integration explicitly links design features with clinical outcome domains and implementation contexts. The contribution is therefore conceptual and integrative, derived from structured synthesis of existing evidence, rather than from primary clinical data generation.

Overall, both AI-enabled and non-AI digital systems appear feasible and potentially effective for supervised clinical rehabilitation and home-based delivery models. Reported adherence rates ranging from 71% to 90% and home-based deployment periods of up to 12 weeks in several included studies support this feasibility [36,37,41]. Notably, the majority of interventions integrated multiple digital components rather than implementing single technologies in isolation, which indicates the importance of the interaction effects between immersive interfaces, adaptive algorithms, and engagement-focused design characteristics. The recent rehabilitation literature indicates that these multi-component digital systems may act synergistically by providing enriched sensorimotor contexts, motivational scaffolding, personalized task adaptation, and thus they have the capacity to increase training dose, training adherence, and learning efficiency over and above single technology solutions [65,66]. The primary theoretical contribution of this review is the presentation of a digital rehabilitation technology ecosystem framework that conceptualizes artificial intelligence, virtual/augmented reality, gamification, sensing technologies, and telerehabilitation as interdependent elements rather than individual tools. The enriched sensorimotor and cognitive contexts within this ecosystem can be facilitated by immersive interfaces [65,67], the mechanisms of gamification can help maintain the motivation and training intensity [68], sensor technologies allow constant monitoring of the performance and remote supervision [66], and AI-based analytics can be used to create the adaptive personalization of the difficulty of the tasks and feedback [69]. By mediating between them, these components create feedback mechanisms that affect the dose of therapy, engagement, and learning effectiveness, thereby providing a coherent conceptual framework to explain heterogeneous digital rehabilitation interventions.

The most common targeted area was upper-limb rehabilitation, and all these improvements were reported in validated scales, including the Fugl–Meyer Assessment (FMA), Action Research Arm Test (ARAT), and Box and Block Test (BBT). The benefits of AI-assisted systems were attained due to real-time monitoring and the adaptive progression of tasks, which helped to facilitate individual therapy courses [34,36,42]. Non-AI digital platforms got similar gains due to the repetition increase, interactive task organization, and user engagement. Mechanistically, it can be seen that synergy is strongest in the situation where immersive VR delivers high-salience sensorimotor contexts, and AI (e.g., reinforcement learning) continuously adjusts task difficulty based on kinematics to form a closed-loop training system, which increases dose and precision in parallel [51,63]. Such interactive activity is supported by the literature on cognitive rehabilitation, where full-immersion VR settings were suggested to improve attention, memory, and executive functioning through the provision of practice conditions that are ecological and rich with feedback [65]. These findings are consistent with overall findings that adaptive feedback, either rule-based or algorithmic, reinforces motor learning and promotes neuroplasticity in all neurological groups [55,70].

Studies using immersive VR, robotic exoskeletons, wearable sensors, and gamified balance environments generally reported improvements in balance and gait outcomes. The use of AI-driven systems to alter task parameters in accordance with user performance was dynamic, unlike non-AI systems that used formal visual and multisensory feedback to encourage postural control [38,41,42]. The synergistic effects are especially evident when interactive telerehabilitation models are used, in which wearable, sensor capture, and remote guidance can be used to maintain real-time feedback loops that maintain training intensity and correct movement quality outside the clinic [66,71]. Balance and gait improvements in older adults and neurological patients have been consistently reported with interactive telerehabilitation interventions that incorporate remote monitoring and remote counseling (through VR, smartphone/tablet applications, or videoconferencing), demonstrating the importance of remote interaction and feedback on mobility outcomes [66]. These results are in line with the latest meta-analytic data proving the better balance improvements through gamified VR than without gamification or traditional rehabilitation systems [54].

Other than stroke, VR-based balance training, exergaming, and feedback-intensive digital pro-programs led to similar benefits in Parkinson’s disease and in multiple sclerosis, with respect to gait, mobility, and postural control [55,56]. Syntheses of evidence in stroke also suggest that VR is more likely to produce effects when used with other traditional modalities, as opposed to being used independently, which suggests an add-on synergy model across conditions and care pathways [72,73]. The wider rehabilitation literature confirms that VR could be used as an immersive modality in the treatment of acquired cognitive disorders, or that multisystem digital environments with high sensory content could concurrently involve motor and cognitive systems [65]. These cross-condition effects indicate that there are shared neuroplastic effects prompted by enriched, adaptive, and feedback-focused online environments, which indicate the overall generalization of digital rehabilitation devices across neurological pathologies.

Gamification emerged as a consistent facilitator of engagement and adherence. Sustained participation was maintained by narrative components, progressive difficulty, incentives, and immediate feedback, and a number of studies have found rates of adherence to be over 80% [33,37,48]. In particular, sensor-based and immersive home environments showed clinically significant improvements in ARAT in up to 84% of users, and motor outcomes that were comparable to those of clinic-based supervised therapy [37,42]. Mood improvements and anxiety reduction were also reported in some of the programs, which is consistent with motivational science evidence that structured gamified effects could improve emotional well-being and persistence [16,74]. Cognitive-oriented platforms showed an extra advantage in the functions of executive, attention, and memory at work. Mechanistically, gamification seems to be synergistic with immersive and adaptive technologies by enhancing intrinsic motivation, intensity of practice, and attention maintenance, which increases the action of training that is task-specific and feedback-driven motor learning [69,75].

The comparison between home-based and clinic-based models yielded significant insights into implementation. Home systems like FitMi (Flint Rehabilitation Devices, Irvine, CA, USA), REWIRE, and BBG attained comparable functional gains to clinic-based interventions and provided the benefit of scaling, access, and lower workload in the therapists [37,39,48]. From a synergy perspective, interactive platforms could be deployed at home and jointly produce a replacement of continuous in-person supervision (i) through engaging task structures, (ii) through remote monitoring/guidance, and (iii) through adaptive progression [66,76]. These results are consistent with previous reviews, which suggest that interactive telerehabilitation is a cost-effective and well-accepted form of rehabilitation [3]. However, clinic-based rehabilitation is still necessary with those people who need additional supervision, sophisticated multisensory settings, robotic support, or dual-task training paradigms [77].

Digital interventions also demonstrated benefits in swallowing rehabilitation. AI-based adaptive systems improved GUSS and FOIS scores while maintaining high adherence levels [36]. Non-AI swallowing tools, including tablet modules and sensory-feedback systems, similarly enhanced accessibility and engagement for patients with mobility constraints. The findings confirm the recommendations of implementing timely, technology-assisted dysphagia rehabilitation to avert complications and enhance the continuity of care [78]. Conceptually, interaction effects are expected when adaptive algorithms personalize task difficulty while gamified feedback sustains practice frequency, enabling higher total swallowing-exercise dose with better tolerability over time [63,79].

Despite promising outcomes, several limitations were evident. Long-term follow-up was largely limited to short- or medium-term assessments (≤3 months), restricting conclusions regarding the sustainability of clinical gains, while formal economic evaluations were rarely reported despite frequent claims related to scalability and accessibility. In addition, heterogeneity in intervention types, populations, AI sophistication, and outcome measures limited comparability and precluded meta-analysis. Sample sizes were frequently small to moderate, often derived from single-center studies, and many trials were underpowered to evaluate interaction effects across combined digital technologies, limiting generalizability. Even though some studies used follow-up measurements of motor, balance, swallowing, or ADL outcomes (4 weeks to 3 months) to prove their maintenance [32,33,34,43,49], longer-term effectiveness remains insufficiently characterized. Evidence from conventional neurorehabilitation suggests that early functional gains may attenuate over time without sustained practice or follow-up interventions, underscoring the importance of evaluating the longer-term durability of digital rehabilitation effects. Several AI-driven systems were validated only in simulated environments, restricting insight into real-world feasibility. Furthermore, reporting of technological implementation was frequently insufficient, particularly for AI-based interventions, with limited description of algorithmic architecture, training data, sensor specifications, or system update mechanisms, constraining reproducibility and clinical interpretability. Consequently, the overall level of evidence varied across outcome domains, with moderate certainty for motor and balance outcomes, and low to very low certainty for usability, adherence, and longer-term outcomes, as reflected in the GRADE assessment.

Although statistical meta-analysis was not feasible due to substantial clinical and methodological heterogeneity, this heterogeneity also highlights important directions for future synthesis. Future studies would benefit from subgroup analyses stratified by (i) neurological condition (e.g., stroke vs. Parkinson’s disease vs. multiple sclerosis), (ii) intervention setting (home-based vs. clinic-based), (iii) technology configuration (single-modality vs. multi-component systems integrating AI, VR, and gamification), and (iv) outcome domain (motor, balance, cognitive, or swallowing). Such stratification may enable more precise estimation of technology-specific effects and clarify which patient subgroups derive the greatest benefit from particular digital rehabilitation approaches.

Assessment of inclusivity-related considerations revealed important gaps across the reviewed literature. Although some studies assessed cognitive or visuospatial status, explicit reporting of platform-level adaptations for individuals with cognitive, visual, or perceptual impairments and the use of inclusive assessment frameworks remained uncommon. Few platforms were designed for individuals with cognitive, perceptual, or visual impairments, limiting inclusivity. Advances in wearable sensor miniaturization and adaptive user interfaces may help address such barriers [80]. While gamification enhances motivation and engagement, overly complex game mechanics may increase cognitive load, particularly among older adults or individuals experiencing fatigue. Integration of user-centered design, adaptive interface, and hybrid human–AI coaching model is to be incorporated in future systems. The use of personalized learning frameworks as evidence indicates the significance of matching the system features with the cognitive and motivational profiles of users to maximize engagement [81,82]. Transparent and explainable AI approaches, supported by standardized tools such as the System Causability Scale, may further enhance clinician trust and facilitate adoption in inclusive rehabilitation settings. Large-scale, longitudinal, and multicenter trials with standardized reporting frameworks are needed to validate these interventions and support clinical translation. Future research studies should extend the follow-up period and assess long-term sustainability based on the new real-world feasibility and follow-up results that have begun to appear in the literature and in AI-based and home-based studies.

## 5. Conclusions

Digital neurorehabilitation technologies incorporating artificial intelligence (AI), gamification, immersive interfaces, and telerehabilitation demonstrate overall feasibility and functional utility across motor, balance, swallowing, and cognitive domains. Adaptive personalization of tasks and automated feedback are more dynamically enabled by AI-driven systems, while engagement and adherence are often facilitated through gamification. Home-based systems demonstrated functional outcomes comparable to clinic-based interventions in selected domains, although clinically supervised environments remain essential for complex or robotics-assisted therapy. Simulation-based studies highlight emerging algorithmic potential, but further clinical validation is required. This review contributes a unified ecosystem framework that synthesizes heterogeneous digital neurorehabilitation approaches and conceptualizes the interaction between AI-driven decision logic, gamification mechanisms, and clinical outcome domains. By integrating clinical evidence with system-level and algorithmic perspectives, the review offers a structured foundation for future engineering development, evaluation, and clinical translation of intelligent neurorehabilitation systems.

### Registration and Protocol

This systematic review was registered in the International Prospective Register of Systematic Reviews (PROSPERO) under registration number CRD420251247889 (Digital and Intelligent Rehabilitation Technologies for Stroke and Neurological Disorders: A Systematic Review of Artificial Intelligence, Virtual Reality, Gamification, and Emerging Therapeutic Platforms in Neurorehabilitation), dated 7 December 2025. The full protocol is accessible via the PROSPERO database. No amendments were made to the original protocol during the course of this review.

## Figures and Tables

**Table 6:** Summary of AI methodologies, inputs, decision logic, and validation context in included studies.

Study (Author, Year)	AI Methodology	Primary Inputs	Algorithm/Decision Logic Reported	Validation Context
Bai (2022) [42]	AI-adaptive VR system	Kinematics, task performance	Supervised learning-based performance monitoring used to adjust task difficulty and progression in real time; adaptation described functionally	Clinical RCT
Zhang (2025) [36]	AI-gamified video-game therapy	Task accuracy, session frequency	Rule-based AI system with adaptive difficulty modulation based on user performance trends across sessions	Clinical RCT
Burdea (2021) [48]	AI-adaptive controller	Error rate, task completion time	Automatic difficulty scaling triggered by performance thresholds; internal decision rules not explicitly specified	Usability study
Lutokhin (2023) [35]	Multimodal AI-assisted rehabilitation	Sensor signals, motor performance metrics	AI-supported personalization combining sensor feedback and performance metrics to modulate training intensity	Clinical RCT
Alsheikhy (2025) [50]	Bi-LSTM + Firefly optimization	Synthetic performance data	Bi-LSTM network predicts task performance; Firefly algorithm optimizes difficulty parameters for personalized VR therapy	Simulation-only
Chen (2024) [15]	GAN-based difficulty generator	Synthetic game/task data	GAN trained to generate task-difficulty levels matching real-data distributions (reported via correlation analysis)	Simulation-only
Pelosi (2024) [51]	Reinforcement learning (Q-learning)	Task success, spatial performance	Q-learning updates task difficulty based on reward signals derived from reaching performance	Simulation/proof-of-concept
de Castro-Cros (2020) [52]	Gamified BCI paradigm	BCI classification output	Decision logic compares gamified vs. non-gamified feedback; no change in classifier accuracy but increased engagement	Pilot/partially simulated

Note: AI = artificial intelligence, VR = virtual reality, BCI = brain–computer interface, GAN = generative adversarial network, Bi-LSTM = Bidirectional Long Short-Term Memory, RCT = randomized controlled trial.

## Data Availability

All data were extracted from the included studies using a standardized data extraction form. No custom analytic code was developed.

## Abbreviations

The following abbreviations are used in this manuscript:

ADLs	Activities of Daily Living
AI	Artificial Intelligence
AR	Augmented Reality
ARAT	Action Research Arm Test
BBT	Box and Block Test
BCI	Brain–Computer Interface
BI	Barthel Index
Bi-LSTM	Bidirectional Long Short-Term Memory
BBG	Balance-Based Games
DL	Deep Learning
FMA	Fugl–Meyer Assessment
FMA-UE	Fugl–Meyer Assessment—Upper Extremity
FOIS	Functional Oral Intake Scale
GAN	Generative Adversarial Network
GRADE	Grading of Recommendations Assessment, Development, and Evaluation
GUSS	Gugging Swallowing Screen
HCI	Human–Computer Interaction
IEEE	Institute of Electrical and Electronics Engineers
κ	Cohen’s Kappa
LMICs	Low- and Middle-Income Countries
ML	Machine Learning
MS	Multiple Sclerosis
PRISMA	Preferred Reporting Items for Systematic Reviews and Meta-Analyses
PROSPERO	International Prospective Register of Systematic Reviews
QoL	Quality of Life
RCT	Randomized Controlled Trial
RL	Reinforcement Learning
RoB 2	Risk of Bias Tool 2
ROBINS-I	Risk of Bias in Non-randomized Studies of Interventions
SCI	Spinal Cord Injury
SWAL-QOL	Swallowing Quality of Life Questionnaire
TBI	Traumatic Brain Injury
VR	Virtual Reality
AR/VR	Augmented Reality/Virtual Reality
Wii Fit	Nintendo Wii Fit Balance Training System
